# Motion patterns in activities of daily living: 3- year longitudinal follow-up after total shoulder arthroplasty using an optical 3D motion analysis system

**DOI:** 10.1186/1471-2474-15-244

**Published:** 2014-07-21

**Authors:** Michael W Maier, Mirjam Niklasch, Thomas Dreher, Felix Zeifang, Oliver Rettig, Matthias C Klotz, Sebastian I Wolf, Philip Kasten

**Affiliations:** 1 Clinic for Orthopedics and Trauma Surgery, Heidelberg University Hospital, Schlierbacher Landstrasse 200a, 69118 Heidelberg, Germany; 2 Orthopaedic Surgery Center (OCC) Tübingen, Tübingen, Germany

**Keywords:** Total shoulder arthroplasty, Shoulder osteoarthritis, Activity of daily living, Outcome, 3D motion analysis, Upper extremity, HUX model, Biomechanic model

## Abstract

**Background:**

Total shoulder arthroplasty (TSA) can improve function in osteoarthritic shoulders, but the ability to perform activities of daily living (ADLs) can still remain impaired. Routinely, shoulder surgeons measure range of motion (ROM) using a goniometer. Objective data are limited, however, concerning functional three-dimensional changes in ROM in ADLs after TSA in patients with degenerative glenohumeral osteoarthritis.

**Methods:**

This study included ten consecutive patients, who received TSA for primary glenohumeral osteoarthritis. The patients were examined the day before, 6 months, and 3 years after shoulder replacement as well. We compared them with a control group (n = 10) without any shoulder pathology and measured shoulder movement by 3D motion analysis using a novel 3 D model. The measurement included static maximum values, the ability to perform and the ROM of the ADLs “combing the hair”, “washing the opposite armpit”, “tying an apron”, and “taking a book from a shelf”.

**Results:**

Six months after surgery, almost all TSA patients were able to perform the four ADLs (3 out of 40 tasks could not be performed by the 10 patients); 3 years postoperatively all patients were able to carry out all ADLs (40 out of 40 tasks possible). In performing the ADLs, comparison of the pre- with the 6-month and 3-year postoperative status of the TSA group showed that the subjects did not fully use the available maximum flexion/extension ROM in performing the four ADLs. The ROM used for flexion/extension did not change significantly (preoperatively 135°-0° -34° vs. 3 years postoperatively 131° -0° -53°). For abduction/adduction, ROM improved significantly from 33°-0° -27° preoperatively to 76° -0° -35° postoperatively. Compared to the controls (118°) the TSA group used less ROM for abduction to perform the four ADLs 3 years postoperatively.

**Conclusion:**

TSA improves the ability to perform ADL and the individual ROM in ADLs in patients with degenerative glenohumeral osteoarthritis over the course of 3 years. However, TSA patients do not use their maximum available abduction ROM in performing ADLs. This is not related to limitations in active ROM, but rather may be caused by pathologic motion patterns, impaired proprioception or both.

## Background

Since the Neer prosthesis was developed in the 1950s, shoulder arthroplasty has advanced considerably. Today, total shoulder arthroplasty (TSA) can significantly improve function in osteoarthritic shoulders [1-4], but a patient’s ability to perform activities of daily living (ADLs) can still remain impaired. Motion analysis of the shoulder is challenging owing to the high range of motion (ROM) of the shoulder. As the clinical gold standard, shoulder ROM is measured by using a goniometer and evaluated by scores such as the Constant score (CS) [5]. Measuring shoulder ROM with a goniometer gives inaccuracies of about 5° to 10° [6]. Furthermore, according to goniometric measurement, it is difficult to distinguish shoulder joint motion from compensatory motion in the trunk and spine. Therefore, a novel model (HUX), as described by Rettig et al. [7], was developed. The HUX model determines the shoulder joint center from the motion data and is dynamically able to capture movement in this calculated shoulder joint center in relation to the torso. With the HUX model, 3D motion can be analyzed in detail after TSA. There is sparse data about the impact of TSA on motion in ADL. Therefore, the purpose of this study was to examine whether TSA is able to restore normal ROM in ADLs in patients with degenerative osteoarthritis of the glenohumeral joint over the course of 3 years.

## Methods

### TSA group

Ten consecutive patients (n = 10; 7 women, 3 men) with a mean age of 65.0 years [SD ± 4.7] and an intact rotator cuff who received TSA for primary glenohumeral osteoarthritis were included in this study. The patients were examined the day before, 6 months and 3 years after shoulder replacement as well. The results of the 6 month follow-up were published in 2010 as ‘pilot study’ [2]. The initial patient cohort at the 6 month follow-up consisted of 13 patients. During the follow-up period, three patients were lost to follow-up, leaving a total of 10 patients for three year evaluation. The dominant side was involved in four cases, the nondominant in six. Six patients were right-hand, four patients left-hand dominant. The same surgeon performed the surgery in all ten patients at the Shoulder and Elbow Section surgery on in the Orthopaedic and Trauma Surgery Clinic of the University Hospital in Heidelberg. All patients received a cemented convex polyethylene glenoid and a cemented humeral stem (Aequalis® Shoulder; Tornier, Lyon, France). The humeral head was anatomically placed in 20° to 30° of retroversion to the transepicondylar axis of the elbow. According to the classification of Walch et al. [8], there were four A2, three B1, and three B2 glenoids. Inclusion criteria for this study were primary or secondary glenohumeral osteoarthritis. Exclusion criteria for this study were stiff shoulder, neurological and muscular diseases, comorbidity rendering the examination impossible, and in addition, lack of verbal communication, fracture prostheses, bipolar prostheses, and rotator cuff failure.

In all shoulders of the TSA group, a deltopectoral approach was used as described by Neer et al. [9]. In no patient was a rotator cuff tear found. After subscapularis tendon detachment and capsular release, the joint was exposed. In all cases, the intraoperative joint status corresponded with the radiographic findings. The biceps tendon was always dissected close to its glenoid attachment and was tenodesed in the bicipital groove. After placing the implant, the subscapularis tendon was repaired by using three to five nonabsorbable tendon-to-tendon sutures. Drains were removed on the first day after surgery. To protect the reconstructed subscapularis tendon, the arm was placed in internal rotation in a shoulder abduction pillow for 4 weeks. Postoperatively, the shoulder was mobilized passively by a physiotherapist for 6 weeks to 60° of flexion and abduction and 0° of external rotation. Patients were asked to support these movements actively. Free ROM was allowed 6 weeks after surgery.

### Controls

The control group included 10 subjects (five women and five men; 20 shoulders) who had no shoulder conditions at the time of the examination upon study entry. No surgery was performed on the controls. Matched controls had a mean age of 64 years [SD 7.3]. All controls were right-hand dominant.

### Joint angle analysis with the HUX model

**Figure 1:**
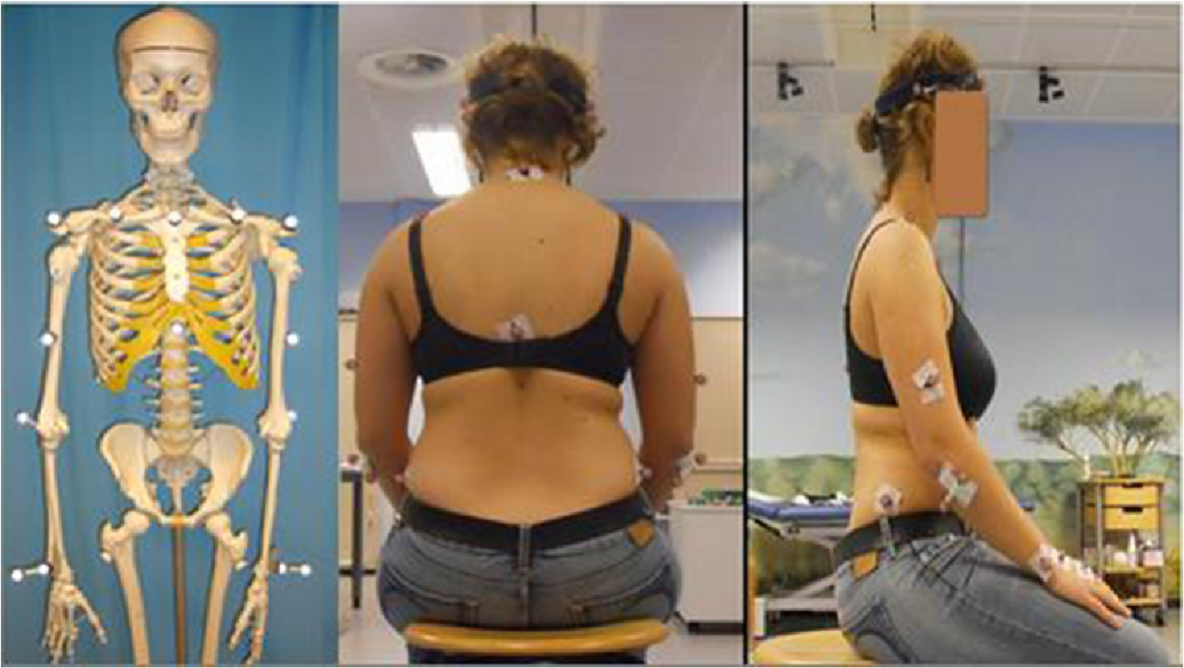
Skeletal model with markers and test person sitting on the chair, prepared with the markers for the 3D motion analysis using the HUX model.

**Figure 2:**
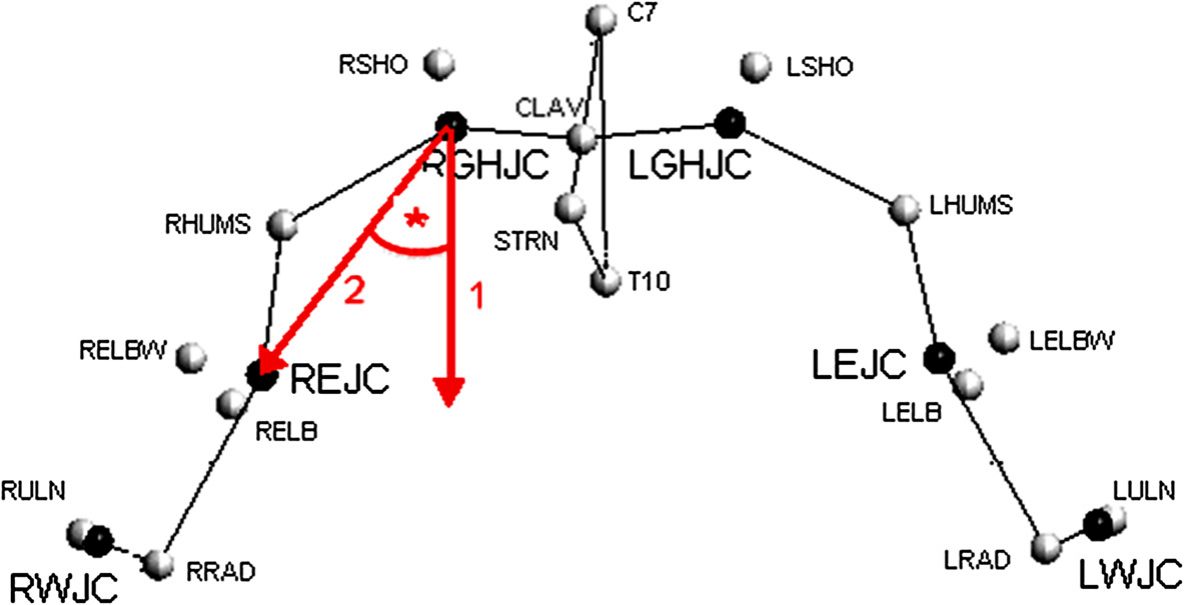
Localization of the glenohumeral joint chenter of rotation (GHJC) and measurement of an angle in the ab-/adduction plane using the HUX model.

All tests for this study were conducted by a single examiner. In accordance with the World Medical Association Declaration, the study protocol was approved by the ethics committee of the Heidelberg medical school (S-305/2007), and informed consent was obtained from all patients and controls. The present study was adhered to the STROBE guidelines. The patients were examined the day before shoulder arthroplasty, 6 months, and 3 years after surgery. The reference data set of the control group was collected once during the first follow-up time of the intervention group. A 12-camera motion analysis system (Vicon 612; Vicon, Lake Forest, USA) operating at 120 Hz was used to observe the motion of the patient. The spatial resolution of the system was about 1 mm. We used the HUX model as described previously by Rettig et al. [7] and applied in some studies [2,10-13]. HUX dynamically defines the functional center of rotation of the shoulder joint, the axis of the elbow joint, and also the center of the elbow joint with a skin “marker set” (Figure 1; we received specific consent to publish from the participant in Figure 1) and seven segments (Figure 2): thorax, clavicles, upper arms, and forearms. Sternoclavicular and glenohumeral joint were treated as a ball-and-socket joint, while the elbow was treated as a hinge joint. Translational degrees of freedom were not considered in any of these joints. The subject was prepared by placing four markers on the trunk as recommended by the International Society of Biomechanics [14] for this measurement. In addition, four markers were placed on each forearm: one at the ulnar and one at the radial styloid process of the wrist. The other two were connected with a wand and placed on the ulna close to the elbow joint [7]. After a static trial, the patient was asked to perform separate movements of elbow flexion/extension, shoulder flexion/extension, and shoulder abduction/adduction to determine the shoulder joint position and the location of the elbow joint axis. Specifically, in these shoulder calibration trials the sternoclavicular joint was considered a cardan joint. Technical coordinate systems for the ulna/forearm, humerus, clavicle, and thorax were not extrapolated by optimization methods as was done for marker clusters [15]. Instead, they were grounded directly on marker trajectories, i.e., the direction vectors between them, using cross-products as demonstrated by Chiari et al. [16].

### Maximum values and ADLs

For flexion/extension and abduction/adduction the corresponding angles between the body’s long axis and the humerus were accounted for (thoracohumeral angle). The body’s long axis is fixed to the thorax-TF; hence, compensatory movement of the thorax can be monitored and distinguished from shoulder movement. To determine the maximum values, the maximum ROM at flexion/extension, abduction/adduction, and also internal and external rotation was dynamically assessed. Angles of flexion/extension and abduction/adduction were expressed as projection angles relative to the proximal anatomical coordinate system. The maximum rotation, defined by the globe convention [17], was measured at 90° degrees of arm abduction to avoid the singularities of the convention for the hanging arm/arm in neutral position. For this, the subject sat on a stool and was instructed to move his/her arm to the respective maximum position without moving his torso. The recordings of ADLs contained the following motions: “combing the hair (cmb)”, “washing the opposite armpit (wsh)”, “tying an apron (aprn)”, and “taking a book from a shelf (shlf)”. Starting from the seated position the subject was asked to carry out these movements by trying not to move the torso. Original position was the static calibration recording and each movement was conducted three times in a row. We calculated an average maximum of the 3 trial maxima. For “combing” the subject held a comb in his/her hand and was asked to move to the forehead for combing from there to the back of the head and finally to go back to the original position. For “washing the opposite axilla” the subject held a washcloth and was asked to move it to the opposite axilla to implement a typical motion of washing there and to return to the original position. For the motion “tying an apron” the subject was asked to move the hand to the back of the torso and then return to the original position. For “taking the book” a height adjustable shelf was used. The height of the shelf was adjusted at forehead level and the book was positioned at the distance of the respective arm length centered to the test person. The subject was then asked to assume the original position place the book in his/her hand, move back to original position with the book, then to put the book back to the shelf and finally to return to the original position without the book. After extracting the motion data using the vicon software (Vicon 612; Vicon, Lake Forest, USA), all calculations were done using Microsoft Excel 2010 software. Statistical analysis was performed using SPSS Version 16.0 (SPSS Inc., Chicago, IL, USA). Group mean values (MV) and standard deviations (SD) were calculated. P values of <0.05 were considered as significant. The distribution of the data was evaluated by using the Shapiro-Wilk test and the homogeneity of variance was assessed using the Levene test. The angle between the long axis of the humerus and the trunk position was determined. The maximum and the minimum angles and the ROM for each task were monitored. The ROMs of the ADL in each plane were compared pre- and postoperative shoulder joint angles were compared by using the Wilcoxon test. As a second outcome measure, differences among these patients and the controls were examined using a Mann–Whitney *U* test.

## Results

Six months after surgery, almost all TSA patients were able to perform the four ADLs (3 out of 40 tasks could not be performed by the 10 patients); 3 years postoperatively all patients were able to carry out all ADLs (40 out of 40 tasks possible) (Table 1).

**Table 1:** Patients in the TSA group who were able to completely perform the activities of daily living before and 6 months and 3 years after surgery

		**Cmb**	**Wsh**	**Aprn**	**Shlf**
TSA n = 10	Pre-op	6	9	9	5
	6 months post	9	10	10	8
	3 years post	10	10	10	10

TSA, total shoulder arthroplasty; Cmb, combing the hair; Wsh, washing the armpit; Aprn, tying an apron; Shlf, taking a book from a shelf.

### ADLs

**Figure 3:**
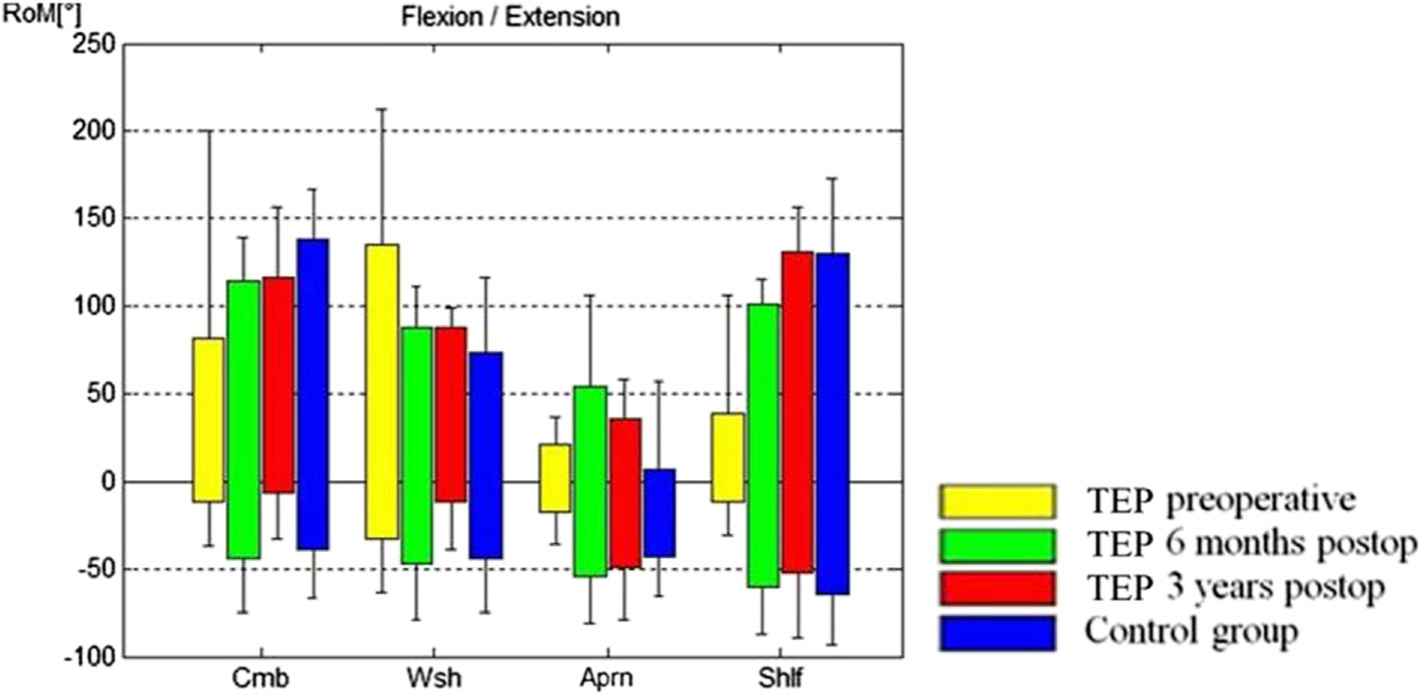
**Range of motion used for performing the ADLs for flexion/extension are shown, comparing the controls with the TEP group preoperatively and 6 months and 3 years postoperatively.** Flexion is marked with positive values, extension with negative values. Cmb, combing the hair; Wsh, washing the armpit; Aprn, tying an apron; Shlf, taking a book from a shelf.

**Figure 4:**
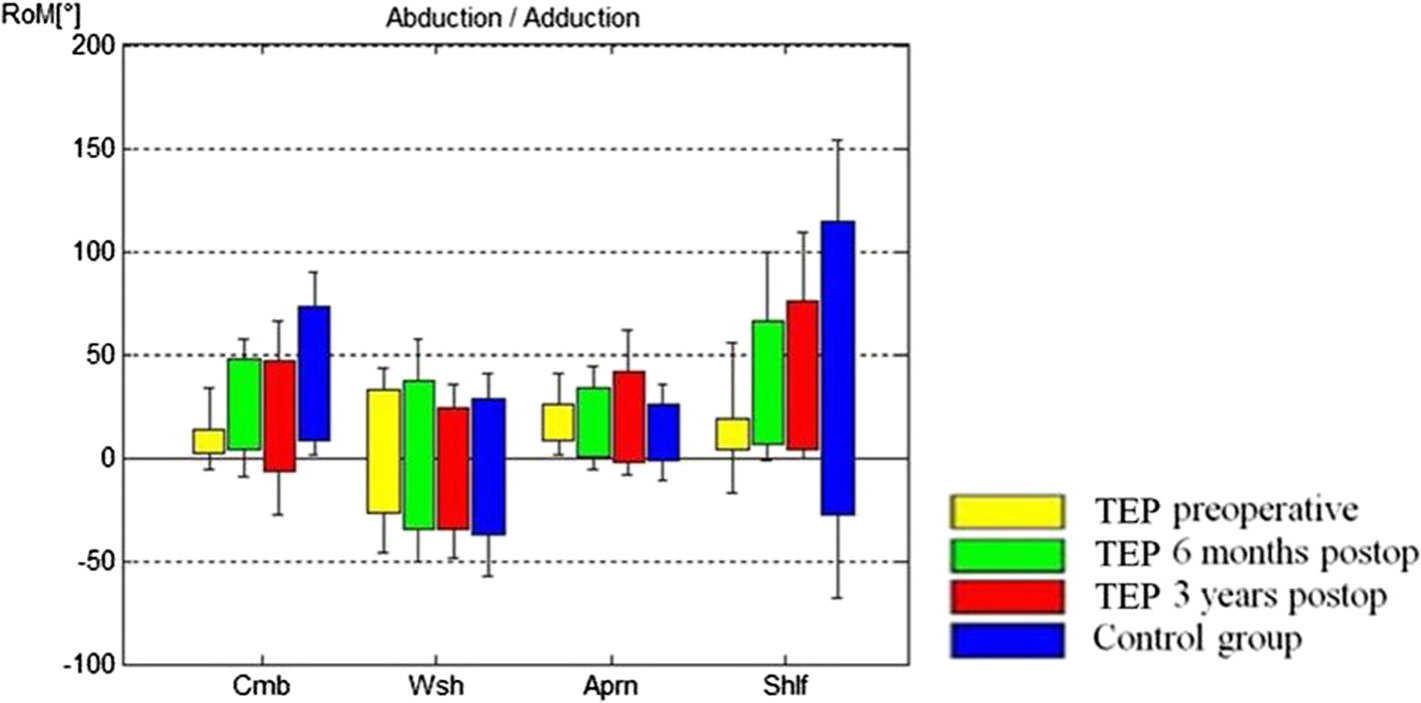
**Range of motion used for performing the ADLs for abduction/adduction are shown, comparing the controls with the TEP group preoperatively and 6 months and 3 years postoperatively.** Abduction is marked with positive values, adduction with negative values. Cmb, combing the hair; Wsh, washing the armpit; Aprn, tying an apron; Shlf, taking a book from a shelf.

In performing the ADLs, comparison of the pre- with the 3-year postoperative status of the TSA group showed that the subjects did not fully use the available maximum flexion, abduction and adduction ROM in performing the four ADLs (Figure 3 and Table 2). In extension, the comparison of maximum available ROM and ROM used performing ADLs showed a significant higher ROM in performing ADLs than in doing the maximum value task (Table 2). The ROM used for flexion/extension did not change significantly (preoperatively 135°-0° -34° vs. 3 years postoperatively 131° -0° -53°). In comparison, the controls used a flexion/extension of 139° -0° -63° to perform the four ADLs (Figure 3). For abduction/adduction, ROM improved significantly from 33°-0° -27° preoperatively to 76° -0° -35° postoperatively (p = 0.024; Figure 4). Comparison of maximum available ROM and maximum used ROM in performing the tested tasks of ADL in the control group showed that they used their full available ROM in abduction and adduction. In extension, the controls showed the same phenomenon of having a higher ROM in performing ADLs than in maximum value task. In flexion, the controls also didn’t use their full available ROM of 168° to perform the four ADLs (Figure 3 and Table 2).

**Table 2:** Comparison of maximum available ROM (Max) and maximum used ROM in performing ADLs (ADL)

	**Patients preoperative**		**Patients 3y postoperative**		**Controls**	
	**Max**	**ADL**	**Max**	**ADL**	**Max**	**ADL**
Flexion	124°	135°	163°	131°	168°	139°
Extension	19°	34°*	35°	53°*	29°	63°*
Abduction	47°	33°	101°	76°	113°	118°
Adduction	11°	27°*	73°	35°	36°	37°

Max = maximum available ROM. ADL = maximum used ROM performing the four ADLs. (*) indicates a significant higher ROM in ADL in comparison to the maximum available static ROM.

### Maximum values

**Figure 5:**
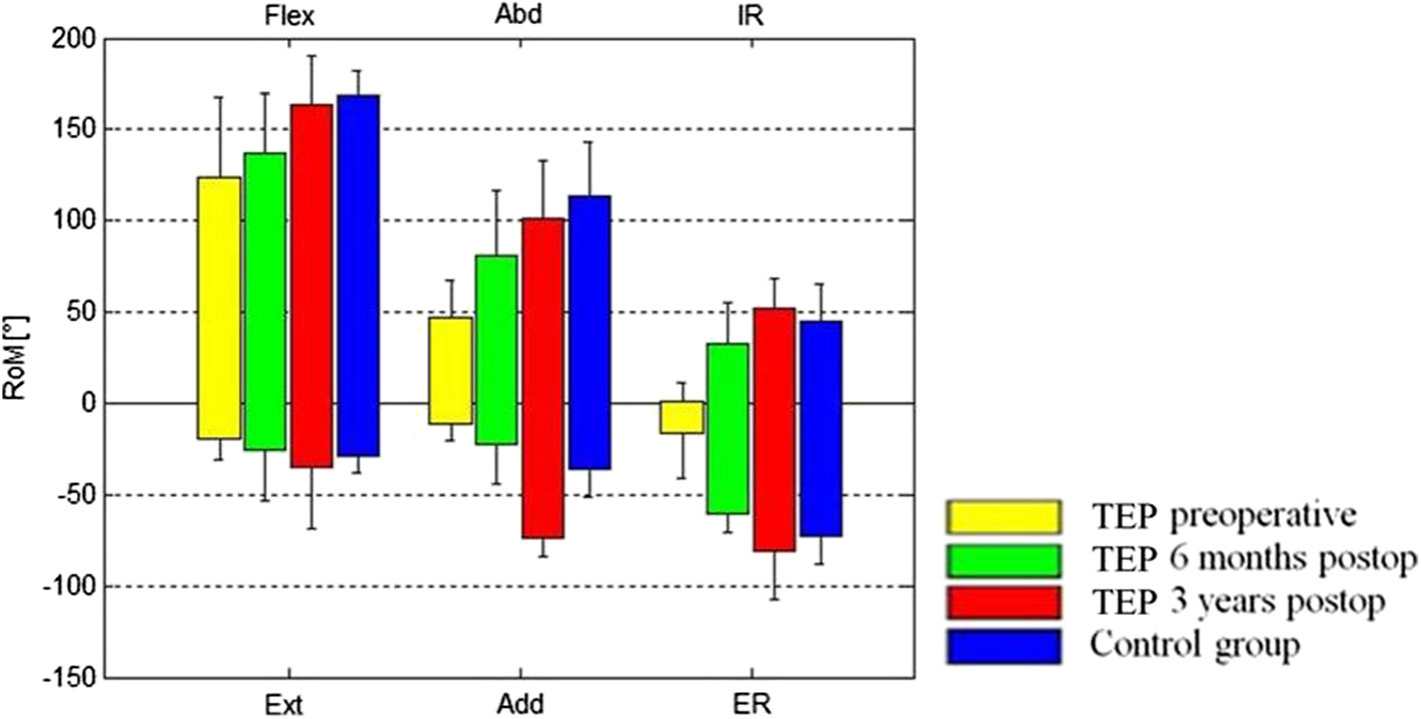
**Comparison of the maximum values of the pre-, 6-month, and 3-year postoperative status in the TEP group with the control group.** Flex, flexion; Ext, extension; Abd, abduction; Add, adduction; IR, internal rotation; ER, external rotation.

Figure 5 shows a comparison of the maximum values from the preoperative and 6-month and 3-year postoperative tests of the TSA group with those of the control group. Comparing the preoperative to the 3-year postoperative ROM in the TSA group, flexion had significantly improved from 124.3° [SD ±43.4°] to 163.7° [SD ±26.7°] (p = 0.047), abduction from 47.1° [SD ±20.6°] to 101.0° [SD ±32.4°] (p = 0.031), adduction from 11.7° [SD ±8.5°] to 73.5° [SD ±10.8°] (p = 0.016), internal rotation from 1.2° [SD ±10.3°] to 51.8° [SD ±17.1°] (p = 0.016), and external rotation from 16.1° [SD ±24.9°] to 81.1° [SD ± 26.8°] (p = 0.016). By trend, extension improved from 19.0° [SD ±11.4°] to 35.2° [SD ±33.8°] (p = 0.219) (Figure 5). A comparison of the 3-year postoperative ROM with the controls showed no significant differences (Figure 5). By trend, the patient group had a greater adduction ROM at 73.5° [SD ±10.8°] than the controls at 36.1° [SD ±15.0°] 3 years postoperatively.

## Discussion

The purpose of this study was to examine whether TSA is able to restore normal ROM in ADLs in patients with degenerative osteoarthritis of the glenohumeral joint over the course of 3 years. We analyzed shoulder motion pre- and postoperatively by three-dimensional video motion analysis using the HUX model as described previously [7] and showed that TSA improves ROM in performing ADLs in patients with degenerative glenohumeral osteoarthritis. Comparison of maximum available ROM and maximum used ROM in performing the tested tasks of ADL in the control group showed that they used their full available ROM in abduction and adduction while in flexion tasks, they didn’t use their fully available ROM to perform the four ADLs (Table 2). In extension, the controls, and the patients’ pre- and postoperative follow-up showed the same phenomenon of having a higher ROM in performing ADLs than in maximum value task. We think that this could be explained by different initial positions. Performing the maximum value task, the subject started from a neutral position while in performing ADLs the reached maximum was independent from a neutral position. In adduction this phenomenon appeared only in the preoperative patient group.

Motion analysis of the upper extremity is challenging because of the great glenohumeral ROM. As the gold standard, maximum ROM is measured with a goniometer which, however, gives inaccuracies of about 5° to 10° degrees and fails to distinguish glenohumeral joint motion from trunk, spine, or scapulothoracical motion [6,18-27]. Patients are asked about their ability to perform ADLs for the CS [5,28,29] but there is no objective assessment of ADLs in clinical routine. The novel HUX model using a 3 D motion analysis system allows for an exact and dynamic capture the movement in the calculated shoulder joint center in relation to the torso without impairment of the motion by e.g. heavy equipment. This means that the success of surgery in ADL can be evaluated better. In the present study, patients who received TSA were examined with respect to maximum glenohumeral ROM plus the ROM in performing ADLs in order to address the following questions: Does the maximum glenohumeral ROM change after TSA? Can shoulder arthroplasty restore a normal ROM in patients with omarthrosis? Are there changes in the ROM used when performing ADLs after shoulder replacement?

The results show that TSA improves the ROM in ADLs but when performing ADLs, TSA patients do not use the maximum available ROM in flexion, abduction and adduction. How can that be explained?

In flexion and adduction the controls also didn’t use more ROM in performing the four ADLs. Therefore we conclud, that it is not necessary to use more flexion and adduction ROM to perfrom the four ADLs. In abduction, however, controls needed 118° of their maximum available 113° to perform the ADLs, while the patients only use 76° of their maximum available 101°. Maybe they used compensatory motion patterns? In the literature, a study of Magermans et al. [30] measured the shoulder motion of healthy subjects with an electromagnetic tracking device while performing ADLs. Their goal was to find the minimal requirements to perform ADL and how these ADL are performed in healthy subjects. For combing the hair, their subjects used at least 73° of glenohumeral elevation comparable to our control group. During arm elevation when combing hair, a large (20–100°) glenohumeral elevation motion was observed by Magermans et al. This shows that the results of the present study are comparable to published data. Veeger et al. [31] hypothesized that the ability to perform an ADL is related to a compensatory movement implementation by means of clavicular retraction. They examined patients after TEP and HEP implantation and divided the patients into two groups according to their ability to comb their hair. Comparable to our results, both patient groups showed limitations in glenohumeral ROM postoperatively as compared to controls. Among patient groups, only axial rotation ROM was different: the ‘Able’ group had a larger external rotational ROM, but less internal rotation. During the ADL ‘combing hair’ the “Able” group appeared to successfully perform the task by applying a larger degree of clavicular retraction. They concluded that functional outcome after arthroplasty is limited due to a lack of glenohumeral ROM but that it is possible to compensate for this restriction by mechanisms such as greater clavicular retraction. The clavicular retraction might be related to a more efficient scapulothoracic motion. In comparison with other published studies [1], our TSA patients showed less postoperative maximum active ROM. The comparative values in the literature were determined by using a conventional goniometer or an electric goniometer, with which it is difficult to adequately distinguish shoulder joint motion from compensatory movements of the trunk. Our measurement method seems to better illustrate pure thoracohumeral joint motion. Under this assumption, we showed that the improved ROM at early follow-up (6 months) after shoulder arthroplasty increased again significantly in the further course (3-year follow-up).

Postoperatively, TSA usually allows each patient to perform all required ADLs painlessly. Considering the maximum ROM in the TSA group, TSA patients were not using their maximum available abduction ROM to perform the ADLs 3 years postoperatively. This is not related to limitations in active abduction ROM, but rather may be caused by impaired proprioception. As the study group showed in a previous investigation [11], proprioception that was measured by an active angle reproduction tended to deteriorate after shoulder arthroplasty. This might be related to the deltopectoral approach that includes divison of the subscapularis muscle and the glenohumeral ligaments and cause alterations in movement patterns. Another explanation could be that the osteoarthritis patients develop impaired motion patterns in ADL that could not be reversed by the sheer possibility to move the arm further because the patients were not sufficiently trained to use the new ROM. A limitation of the study is the relatively low number of subjects. This might disguise further significant changes. We did not examine the state of rotator cuff at the last follow-up. This parameter could also influence the result of the study. The study setting is, however, very elaborate and time consuming, and cannot be used in a routine follow-up.

## Conclusion

TSA improves the ROM in performing ADLs in patients with degenerative glenohumeral osteoarthritis. TSA patients do not use their maximum available abduction ROM in performing ADLs. This is not related to limitations in active ROM, but rather may be caused by pathologic motion patterns, impaired proprioception or both.

## Abbreviations

TSA: Total shoulder arthroplasty; ADL: Activity of daily living; ROM: Range of motion; HUX: Heidelberg Upper Extremity Model; Cmb: Combing the hair; Wsh: Washing the opposite armpit; Aprn: Tying an apron; Shlf: Taking a book from a shelf; SD: Standard deviation.

## Competing interests

The authors declare that they have no competing interests. All authors, their immediate family, and any research foundation with which they are affiliated did not receive any financial payments or other benefits from any commercial entity related to the subject of this article.

## Authors’ contributions

MM made the concept and design, and performed the acquisition of data, analysis and interpretation of data and wrote the manuscript. MN participated in data acquisition, interpretation of data, statistics and helped prepare the manuscript. TD made substantial contributions to data acquisition and interpretation from the institutional motion analysis laboratory. FZ did the surgery, participated in the interpretation of data and OR was the technical developer of the Heidelberg Upper Extremity model and participated in the interpretation of data. MK participated in data acquisition from the institutional motion analysis laboratory and statistics. SW was involved in the development of the motion analysis model, data acquisition, data interpretation and manuscript approval. PK did the design of the study, data acquisition, interpretation of data, manuscript preparation and gave final approval of the version to be published. All authors read and approved the final manuscript.

## Pre-publication history

The pre-publication history for this paper can be accessed here:

http://www.biomedcentral.com/1471-2474/15/244/prepub
